# The Essential Role of ClpXP in *Caulobacter crescentus* Requires Species Constrained Substrate Specificity

**DOI:** 10.3389/fmolb.2017.00028

**Published:** 2017-05-09

**Authors:** Robert H. Vass, Jacob Nascembeni, Peter Chien

**Affiliations:** ^1^Molecular and Cellular Biology Graduate Program, University of MassachusettsAmherst, MA, USA; ^2^Department of Biochemistry and Molecular Biology, University of MassachusettsAmherst, MA, USA

**Keywords:** CLPX, CLPP, ClpXP, *Caulobacter crescentus*, ATP-Dependent Proteases

## Abstract

The ClpXP protease is a highly conserved AAA+ degradation machine that is present throughout bacteria and in eukaryotic organelles. ClpXP is essential in some bacteria, such as *Caulobacter crescentus*, but dispensible in others, such as *Escherichia coli*. In *Caulobacter*, ClpXP normally degrades the SocB toxin and increased levels of SocB result in cell death. ClpX can be deleted in cells lacking this toxin, but these Δ*clpX* strains are still profoundly deficient in morphology and growth supporting the existence of additional important functions for ClpXP. In this work, we characterize aspects of ClpX crucial for its cellular function. Specifically, we show that although the *E. coli* ClpX functions with the *Caulobacter* ClpP *in vitro*, this variant cannot complement wildtype activity *in vivo*. Chimeric studies suggest that the N-terminal domain of ClpX plays a crucial, species-specific role in maintaining normal growth. We find that one defect of *Caulobacter* lacking the proper species of ClpX is the failure to properly proteolytically process the replication clamp loader subunit DnaX. Consistent with this, growth of Δ*clpX* cells is improved upon expression of a shortened form of DnaX *in trans*. This work reveals that a broadly conserved protease can acquire highly specific functions in different species and further reinforces the critical nature of the N-domain of ClpX in substrate choice.

## Introduction

Energy dependent proteolysis is a cellular process that maintains protein homeostasis, quality control, and allows for temporal changes in protein concentration required for cell signaling (Sauer and Baker, 2011). ClpXP is a conserved protease complex that performs highly targeted degradation. ClpXP is a two-part protease system consisting of a regulatory element (ClpX) and peptidase (ClpP) and is present throughout biological systems, ranging from bacteria to eukaryotic organelles. ClpX requires the use of ATP to self oligomerize, recognize, and unfold target proteins. The unfoldase has two main functions; (1) recognize substrates and (2) translocate them into the ClpP pore for degradation. The AAA+ domain of ClpX contains the Walker motifs that bind/hydrolyze ATP and the central pore loops required for substrate engagement (Baker and Sauer, 2012). An additional unique feature of ClpX is its N-domain, which is needed for recognition of some protease substrates. Regardless of how they are recognized, all substrates must be translocated to ClpP. Therefore, ClpX must interact effectively with ClpP to realize the full potential of this protease (Singh et al., 2001; Joshi et al., 2004).

The ClpX unfoldase must regulate which substrates are targeted for destruction by the ClpP chamber (Baker and Sauer, 2012). For example, in the bacterium *Caulobacter crescentus*, ClpX activity responds to cell cycle cues and stresses to meet the proteolytic demands as needed (Jenal and Fuchs, 1998; Smith et al., 2014; Williams et al., 2014; Joshi et al., 2015; Lau et al., 2015; Vass et al., 2016). To accomplish these different proteolytic tasks, ClpXP recognizes substrates using both simple degradation tags (degrons) and with the assistance of adaptor proteins that promote degradation of new substrate pools in a ClpX N-domain dependent manner (Baker and Sauer, 2012; Vass et al., 2016). One instance of this complex regulation is during trans-translation, where the rescue of stalled ribosomes is accompanied by the appending of the SsrA peptide, which is recognized by the ClpXP protease, to improperly translated polypeptides leading to their destruction (Tu et al., 1995; Keiler et al., 1996; Gottesman et al., 1998). Although this base recognition is independent of the ClpX N-domain, the SspB adaptor can further improve degradation of SsrA-tagged substrates by binding the N-domain of ClpX (Levchenko et al., 2000).

The ClpXP complex is not essential in all organisms. For example, ClpXP is dispensable in *Escherichia coli* (Gottesman et al., 1993), but is required in *C. crescentus* (Jenal and Fuchs, 1998; Osteras et al., 1999). Recent work points to a critical role of ClpXP in *Caulobacter* through the essential processing of the replication clamp loader subunit DnaX, driving cell cycle progression, and destruction of the toxin SocB—processes that are absent in *E. coli* (Aakre et al., 2013; Vass and Chien, 2013; Joshi and Chien, 2016). Interestingly, despite high homology, the *E. coli* ClpX cannot complement the essential ClpX function in *Caulobacter* cells (Jenal and Fuchs, 1998; Osteras et al., 1999). Here, we use chimeric variants of ClpX to determine which features of this protease are important for either species-specific or species-nonspecific activity. We find that the N-domain of ClpX plays an especially important role in regulating essentiality in *Caulobacter*, but that expression of a non-complementing ClpX provides benefit during cell growth. Together, our work demonstrates how ClpXP specificity regulates species-specific responses in a bacterium where this protease is essential.

## Results

### *Escherichia coli* ClpX forms an active protease with *Caulobacter* ClpP *in vitro*

**Figure 1:**
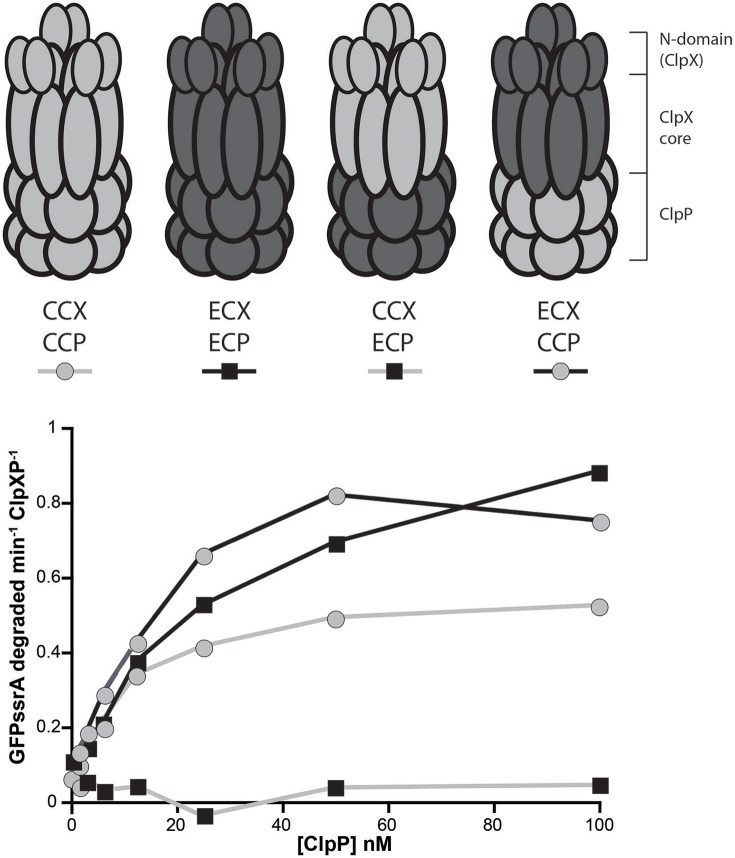
*****Escherichia coli*** ClpX forms an active protease with ***Caulobacter crescentus*** ClpP**. Degradation reactions of 1 μM GFP-ssrA by 50 nM ClpX_6_ with varying concentrations of ClpP_14_ as shown on the x-axis. Initial rates of degradations are plotted as a function of ClpP_14_ concentration.

Prior work suggests that the *E. coli* ClpX cannot substitute for ClpX in *Caulobacter* (Osteras et al., 1999). What are the differences between *E. coli* ClpX (ECX) and *Caulobacter* ClpX (CCX) that restrict essentiality in *Caulobacter*? An alignment of ECX to CCX protein sequences reveals high identity (68%) and a total homology of ~90% (Supplemental Figure 1). We sought to understand why these enzymes do not substitute for each other despite their high similarity. A simple explanation for the inability of ECX to complement in *Caulobacter* may be an inability for ECX to bind with the *Caulobacter* ClpP and form an active protease. We tested this hypothesis by monitoring ClpXP dependent degradation of GFP-ssrA where loss of fluorescence occurs when ClpX successfully delivers substrate to ClpP (Figure 1).

Both ECX and CCX are able to deliver substrate to *Caulobacter* ClpP (CCP), while only ECX can recognize and degrade GFPssrA together with *E. coli* ClpP (ECP, Figure 1). By titrating ClpP, we can derive an effective binding of ClpX to ClpP as a measure of protease activation (*K*_*activation*_) and find similar strengths of interactions between ClpX and ClpP in those combinations that result in an active protease (Table 1). This suggests that both ECX and CCX associate similarly with CCP. Note that the CCX + ECP combination fails to degrade GFPssrA (Figure 1), but because this combination is not germaine to this current work, we did not further explore this observation in this manuscript. Our major conclusion from this characterization is that it seems that ECX forms a productive protease with CCP, therefore the failure of ECX to replace CCX *in vivo* (Osteras et al., 1999) likely stems from a failure to maintain a particular substrate degradation profile rather than a failure of protease assembly. We decided to capitalize on this difference in activity to explore how species-specific elements of ClpX are required in different bacteria.

**Table 1:** **Apparent binding constants between ClpX and ClpP using K_**activation**_ as a proxy**.

**Protease composition**	**K_activation_ (nM)**	**Maximum rate (/min/ClpXP)**
CCX + CCP	9.1 ± 1.1	0.57 ± 0.02
ECX +CCP	12.1 ± 3.3	0.92 ± 0.08
ECX + ECP	24.1 ± 5.5	1.06 ± 0.09
CCX+ ECP	N/D	N/D

*Values are derived from fitting degradation data for active proteases from Figure 1 to a first-order binding equation (degradation rate = maximum rate/(K_activation_+ [ClpP])). Because CCX + ECP does not degrade GFPssrA, we did not fit this data (N/D)*.

### The N-domain of *Caulobacter* ClpX harbors an essential species-specific function

Although the ClpX pore is critical for substrate recognition, the ClpX N-domain provides additional specificity, often driven upon the binding of the N-domain by adaptor proteins that aid in degradation of substrates. We speculated that the ClpX N-domain contains species-specific motifs that provide for the essential activity in *Caulobacter*. Because ECX could form an active protease with CCP *in vitro*, we inferred that the AAA+ domain of ECX was sufficient to interact with CCP, as the N-domain is dispensable for the ClpX-ClpP interaction (Singh et al., 2001). Therefore, we used this system to determine how different variants and chimeras of ECX or CCX could support viability in *Caulobacter*.

**Figure 2:**
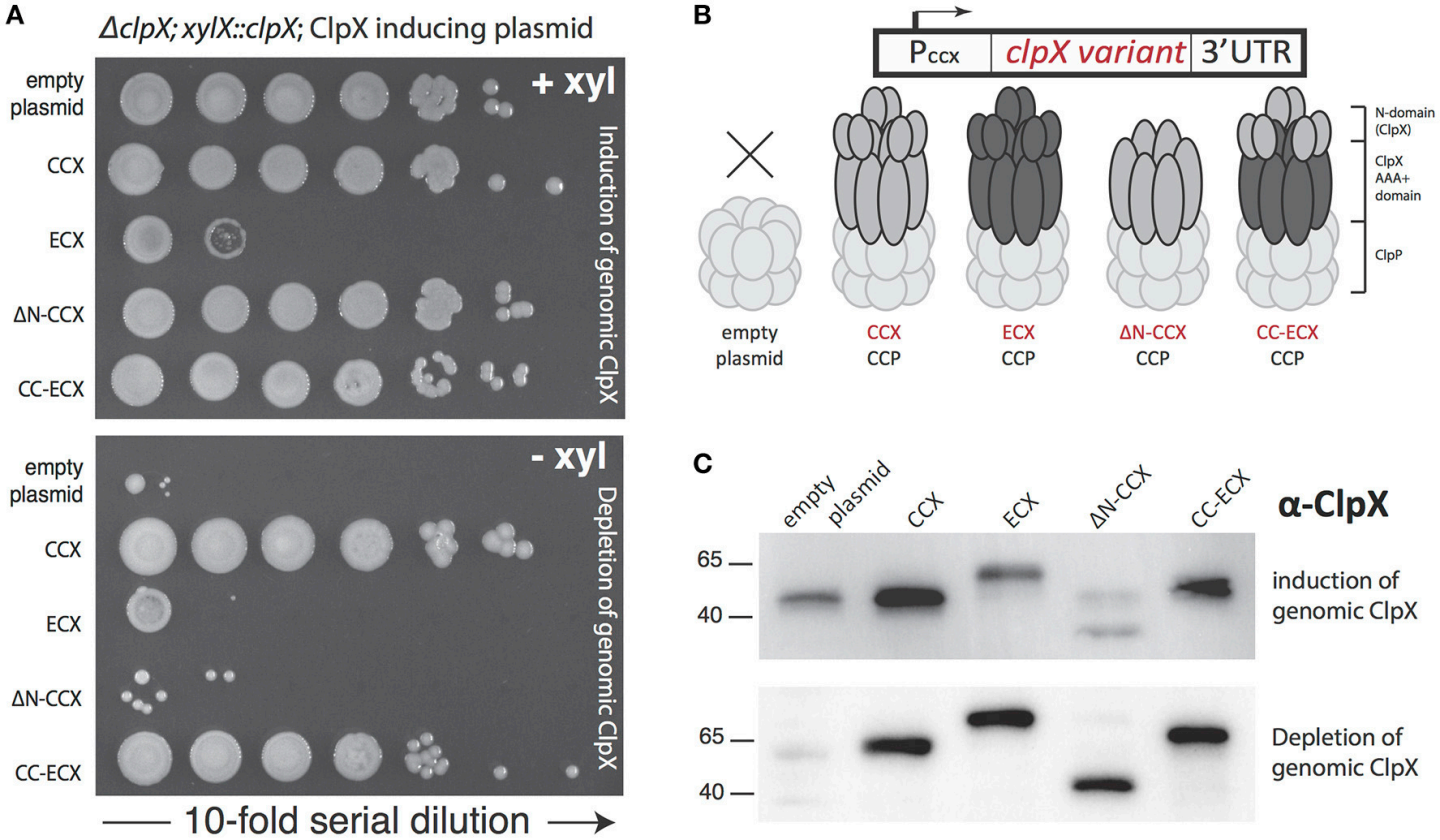
**Only ClpX that contains the ***Caulobacter*** N-domain is able to support viability. (A)** Depletion of genomic ClpX by removal of xylose provides a background to test if plasmid encoded, constitutively expressed ClpX variants are capable of complementing viability. Survival seems restricted to constructs that contain the *Caulobacter* N-domain (see Supplemental Figure 3 for replicate). **(B)** Plasmid constructs contain the *Caulobacter* ClpX promoter to drive constitutive expression of the ClpX variants. **(C)** Monitoring of ClpX levels by Western illustrates the successful depletion of genomic ClpX and the presence of plasmid expressed ClpX variants.

We expressed different ClpX variants in a strain background where the endogenous ClpX could be depleted (Osteras et al., 1999). Similar to what had been reported previously (Osteras et al., 1999), expression of ECX from a plasmid failed to complement, while similar expression of CCX restored growth (Figure 2A). Expression of a CCX lacking the N-domain (ΔN-CCX) was also unable to support viability (Figure 2A; Bhat et al., 2013). Interestingly, a chimeric construct consisting of the N-domain of CCX fused to the AAA+ domain of ECX (CC-ECX) was able to restore viability in this background (Figures 2A,B). Western analysis confirms the expression of the appropriate constructs and the depletion of the endogenous ClpX (Figure 2C). The presence of ECX also affects normal *Caulobacter* growth even in the presence of CCX (Figure 2A; +xyl), which we speculate may be due to ECX binding to CCP and disrupting the formation of productive CCX+CCP complexes. Taken together with our *in vitro* work (Figure 1), our data suggests that the CCX N-domain is required for identification of substrates and proper degradation, which is ultimately needed for *Caulobacter* survival.

### Bypassing the essential requirement for ClpX reveals nonessential proteolysis important for growth

Recent work suggests that the regulated destruction of the SocB toxin by the ClpXP protease via the adaptor SocA justifies the essential need for ClpX in *Caulobacter* (Aakre et al., 2013). In this model, depletion of ClpXP results in accumulation of the SocB toxin and cell death. It is possible that the CCX N-domain contains unique regions needed for interacting with the SocA adaptor to promote SocB degradation. If so, these regions are either absent in ECX or they are masked, which would explain the finding that ECX fails to complement viability (Figure 2A). An alternative model is that the ECX engages inappropriately with other target proteins, which results in cell death due to prolific degradation. We sought to distinguish between these models by taking advantage of strains where *socB* is deleted.

**Figure 3:**
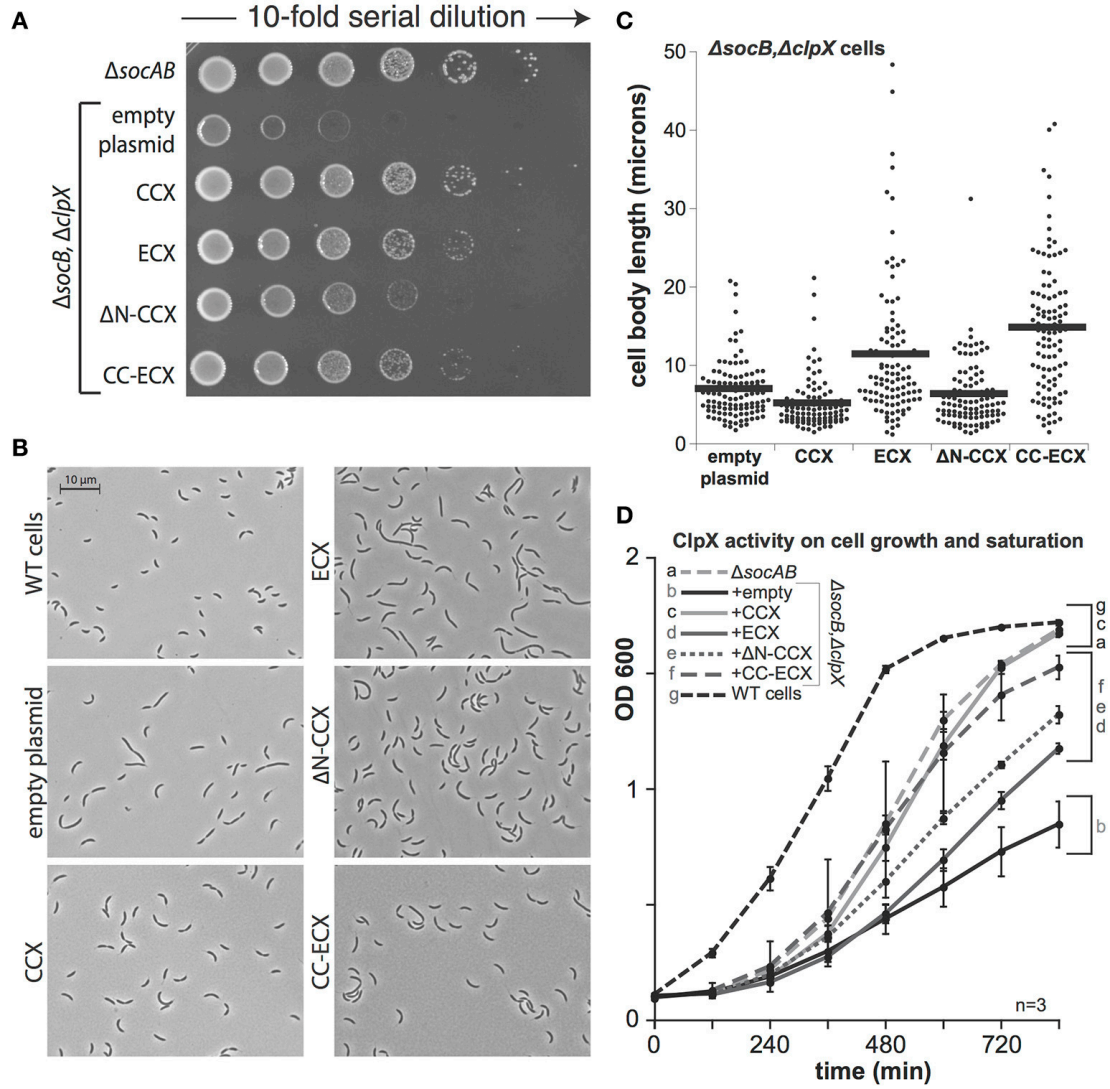
**The presence of ClpX shapes normal growth in ***Caulobacter***. (A)** The presence of any ClpX variant improves growth when both SocB and ClpX are absent (see Supplemental Figure 3 for replicate). **(B)** Microscopic examination shows differences in cell length and morphology dependent on ClpX variant. **(C)** Quantification of cell length (in microns) for strains shown in B, *n* > 100. (black bars denote mean length in microns for each strain). **(D)** Expression of any ClpX improves cell mass accumulation (*n* = 3, error bars are standard deviation). Restoration of wildtype growth requires both the *Caulobacter* N-domain and AAA+ domain (a,c), but expression of any ClpX variant results in partial growth restoration (d,e,f) compared to no ClpX (b).

In cells lacking SocB, *clpX* could be deleted, but these cells are abnormal and show poor viability upon plating (Figure 3A). As expected, expression of CCX restored viability in a dilution-plating assay (Figure 3A). However, in contrast to prior observations (Osteras et al., 1999, Figure 2A), expression of ECX complements growth (Figures 3A,D). The ΔN-CCX construct also improves viability, though less effectively than variants of ClpX with an N-domain (Figures 3A,D). Microscopy studies reveal that expression of CCX in Δ*clpX*Δ*socB* cells restores normal morphology and cell length (Figures 3B,C). Interestingly, although expression of ECX restores viability, cell morphology and cell length are still dramatically perturbed (Figures 3B,C). This perturbation is also seen with expression of the chimeric CC-ECX construct (Figures 3B,C), suggesting that species-specific differences in the ClpX AAA+ domain are responsible for these changes in cell morphology. Consistent with this interpretation, expression of the ΔN-CCX restores cell length more fully than either of the constructs containing the ECX AAA+ domain (Figures 3B,C). Thus, it seems that there are species-specific N-domain dependent and AAA+ domain-dependent substrate recognition profiles that both contribute to the role of ClpX in *Caulobacter*.

### Species-specific processing of DnaX is needed for robust growth

Given the species-specific nature of the phenotypic complementation, we next explored the molecular consequences of ClpX variant expression.

**Figure 4:**
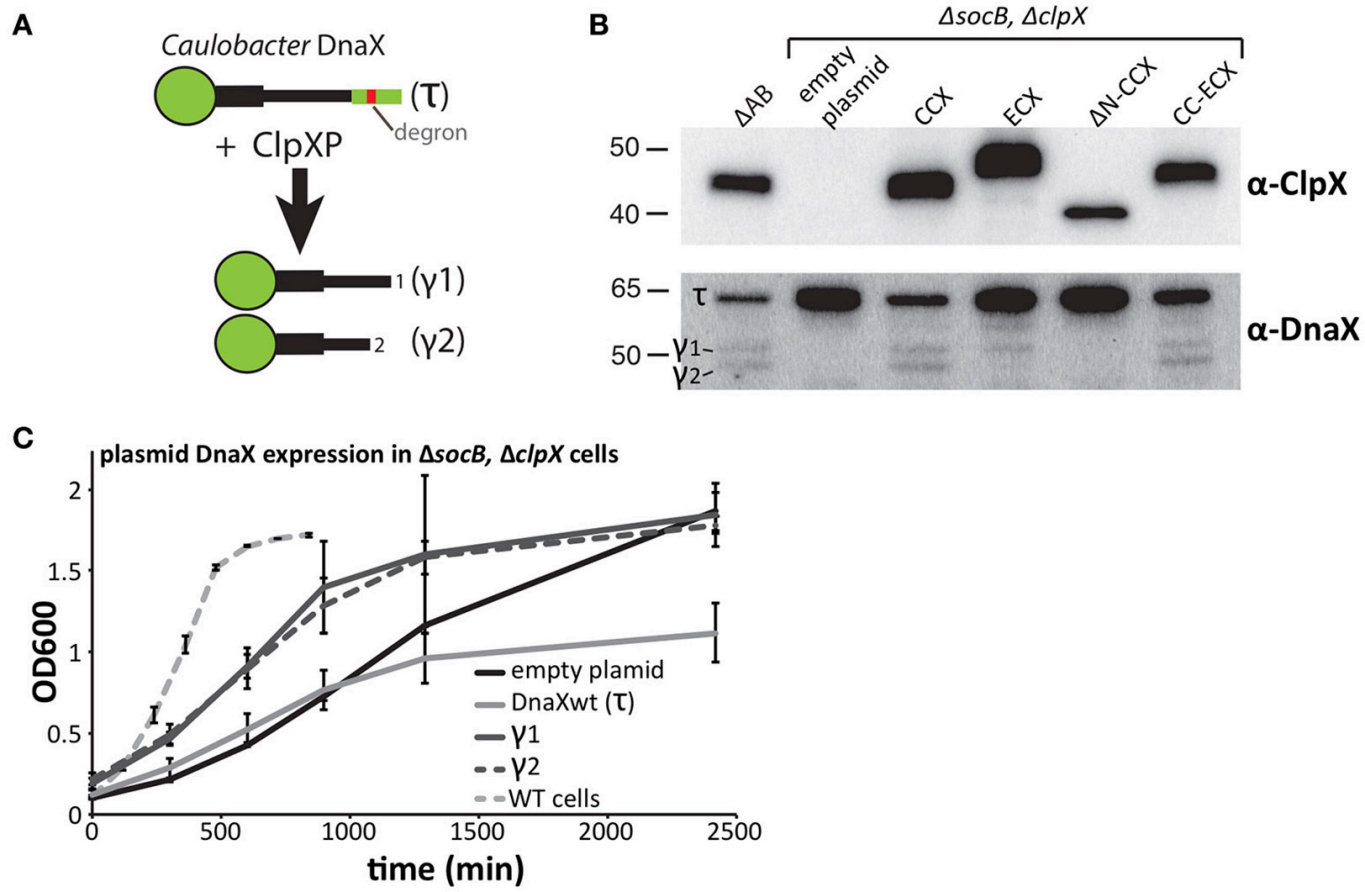
**Processing of DnaX improves ***Caulobacter*** growth. (A)** Processing DnaX τ into either γ1 or γ2 requires ClpXP recognition of the degron and release of stable fragments. **(B)** Cells that lack ClpX contain only full length DnaX. Expression of ClpX variants harboring the *Caulobacter* N-domain result in processing of DnaX. Expression of ECX result in aberrant fragment formation while expression of ΔN-ClpX fails to produce DnaX fragments (See Supplemental Figure 4 for replicate blots). **(C)** In cells that lack both SocB and ClpX, supply of either γ1 or γ2 *in trans* increases the *Caulobacter* growth. Additional expression of wildtype DnaX results in a lower cell mass at saturation (n=3; error bars represent standard deviation).

DnaX is a subunit of the replication clamp loader complex that is responsible for sliding clamp dynamics during replication and DNA damage responses (Kelch, 2016). In *Caulobacter*, full length DnaX (also called τ) is processed by the ClpXP protease to generate shorter fragments (γ1 and γ2) that are critical for survival and a robust DNA damage response (Figure 4A; Vass and Chien, 2013). Because Δ*socB* cells can tolerate the loss of ClpX, we examined the levels of DnaX in this background. In line with our expectations, DnaX was not processed in cells lacking ClpX (Figure 4B). Previous *in vitro* work suggested that the N-terminal domain of ClpX plays an essential role for proteolytic recognition of DnaX (Vass and Chien, 2013) and, consistent with this model, cells expressing ΔN-CCX fail to process DnaX. However, this *N-domain* dependence is species-specific, as cells expressing ECX also do not correctly process DnaX, resulting in loss of the shortest (γ2) DnaX and accumulation of full length DnaX (Figure 4B). The ECX AAA+ domain is able to process DnaX correctly as expression of the CC-ECX chimeric ClpX, which contains the ECX AAA+ domain, is sufficient to restore the production of both normal DnaX fragments. Therefore, species-specific combinations of the N-domain and the ClpX AAA+ domain are needed for normal processing and degradation of DnaX.

Previously, we showed that DnaX processing is essential for wildtype growth (Vass and Chien, 2013), however Δ*socB*Δ*clpX* strains are viable even though DnaX is not processed in this background (Figure 4B). Given the sickness of these cells, we asked if expression of the γ-fragments of DnaX could improve growth in these strains. Consistent with a critical need for DnaX isoforms, we found expression of either γ1 or γ2 DnaX increased growth rate in liquid cultures, compared to the empty plasmid control (Figure 4C). Curiously, expression of full length DnaX (which only generates τ in this ClpX-free strain) inhibits growth and reduces density at saturation suggesting that an excess of τ is toxic. Despite the clear improvement in growth, the doubling time of γ1 or γ2 expressing strains is still ~9–10 h (Figure 4C), substantially longer than the ~90 min doubling time of wildtype *Caulobacter* in these conditions. Therefore, there must be additional non-essential aspects of ClpXP degradation that promote normal robust growth.

### Cell cycle adaptors do not rely on species restricted interactions with ClpXP

*Caulobacter* growth and development relies on adaptors that interact with the ClpX N-domain (Aakre et al., 2013; Smith et al., 2014; Lau et al., 2015). The ECX AAA+ domain is active (Figure 1) but the ECX variant results in a DnaX distribution different from CCX (Figure 4B). Therefore, we next asked if adaptor mediated degradation was altered in strains expressing ECX.

**Figure 5:**
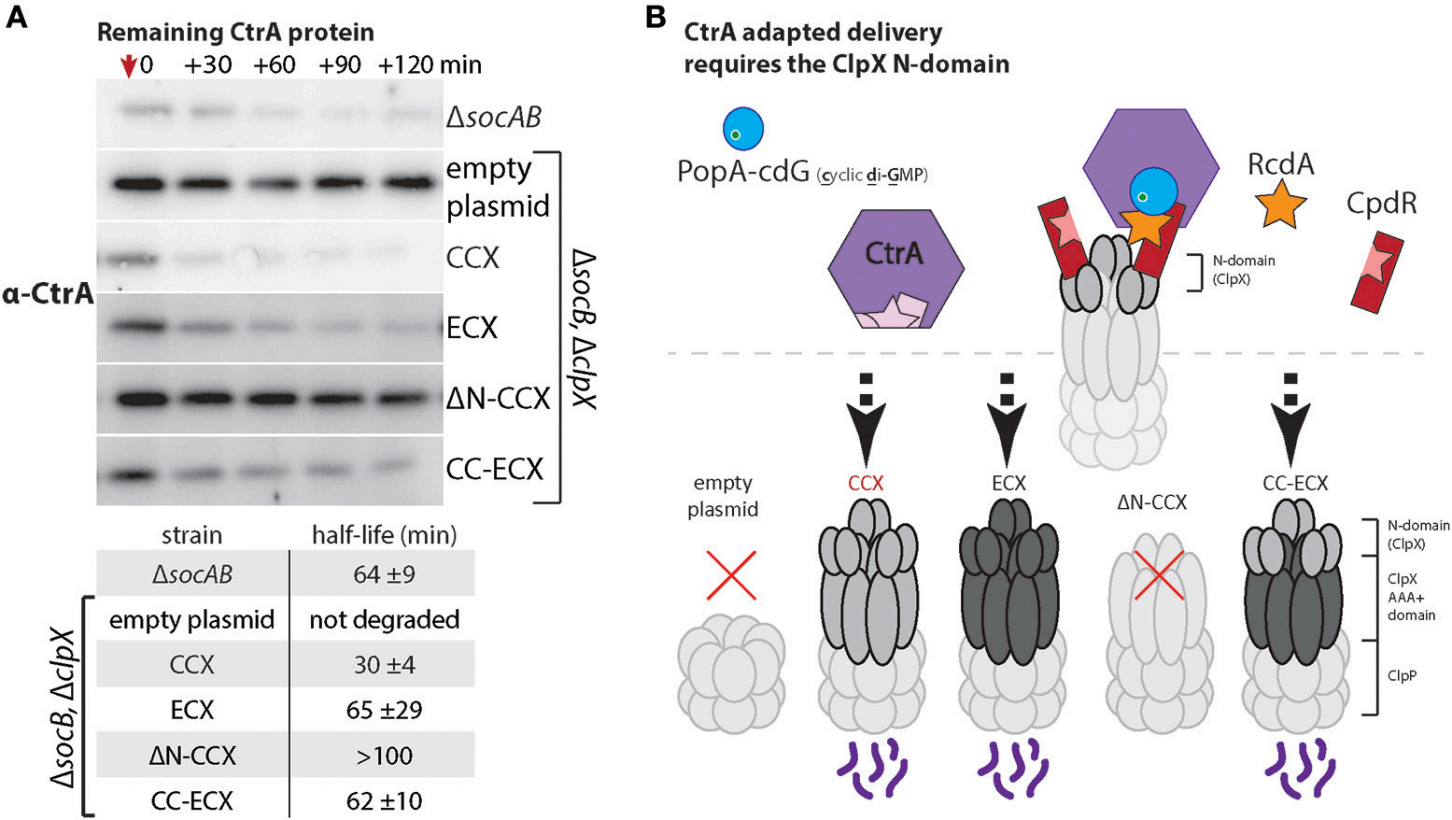
*****Escherichia coli*** ClpX can use ***Caulobacter*** adaptors to effectively degrade CtrA. (A)** Measuring CtrA levels after addition of chloramphenicol (at red arrow) reveals that both CCX and ECX N-domains can support CtrA proteolysis. The table includes half-lives and standard deviation averaged over three individual experiments (see Supplemental Figure 5 for replicate blots and quantification). As expected, cells lacking ClpX or expressing only ΔN-ClpX fail to degrade CtrA (Smith et al., 2014). **(B)** Model showing how rapid degradation of CtrA requires the combination of CpdR, RcdA, and PopA with cdG (cyclic di-GMP) to interact with the ClpX N-domain.

CtrA is a master regulator and replication inhibitor in *Caulobacter* that must be degraded during the transition from the swarmer to stalked cell to promote replication and developmental changes (Jenal and Fuchs, 1998; Wortinger et al., 2000). Degradation of the CtrA protein is an excellent model for N-domain dependent delivery as this process requires a multi-adaptor hierarchy consisting of CpdR, PopA, and RcdA (Taylor et al., 2009; Smith et al., 2014; Joshi et al., 2015; Lau et al., 2015). By monitoring the adaptor-dependent delivery of CtrA we could explicitly test if the ECX N-domain was capable of supporting these adaptor interactions. As a read out of CtrA degradation, we used Western blotting to monitor levels of CtrA following inhibition of protein synthesis upon addition of chloramphenicol. As anticipated, cells containing the CCX N-domain (CCX, CC-ECX) can degrade CtrA while cells without ClpX or expressing ΔN-CCX are unable to degrade CtrA robustly (Figure 5A). Cells expressing ECX as the only ClpX variant exhibit CtrA degradation similar to wildtype (Figure 5B). Thus, the N-domain of ECX is able to support degradation through the adaptor hierarchy found in *Caulobacter*.

Our working model is that ECX fails to degrade the SocB toxin because the N-domain of ECX fails to bind the SocA adaptor (Figure 2). However, the N-domain of ECX appears fully competent to interact with the cell cycle adaptor hierarchy (Figure 5). Because adaptor-dependent delivery requires unique interactions supplied by the N-domain and contacts with the ClpX AAA+ domain, our work reveals a complexity in this regulation that results in both species-specific and species-nonspecific recognition of protease substrates.

## Discussion

The presence of the ClpX unfoldase in all bacteria is likely due to a need for its protease activity. Given the similarity between orthologs, it is perhaps not surprising that many species of ClpXP can universally recognize some substrates based on conserved sequence or structural degrons, such as SsrA-tagged proteins. Increasing the versatility of ClpX activity therefore requires additional elaboration of ClpX-substrate interactions. Adaptors can fill this role, but are not the only method of diversifying substrate recognition.

**Figure 6:**
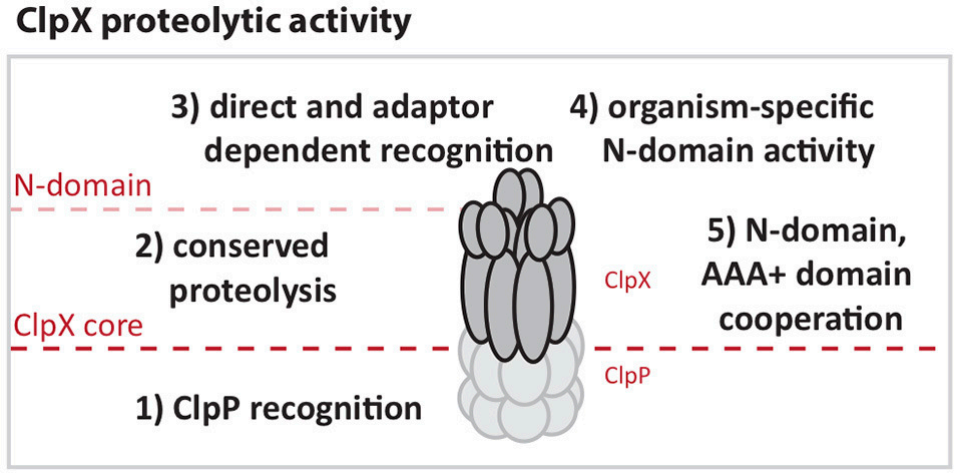
**ClpX activities are defined by N-domain and AAA+ domain functions**. (1) ClpX must interact with ClpP to enable proteolysis so that (2) Substrates directly targeted to or that have engaged the ClpX pore can be degraded. (3) Additional contact and selectivity by N-domain increases regulation through recognition and adapted delivery that can enhance stringency or change ClpXP substrate load. (4) Certain portions of the N-domain contain species-specific regions that target unique substrates. (5) Chimeric studies suggest that cooperation between substrate recognition by the N-domain and AAA+ domain have undergone optimization for species-specific activity.

Our comparison of *E. coli* and *Caulobacter* ClpX reinforces the working model that the most conserved regions of the ClpX AAA+ domain support functions required for all protease activity, such as ATP hydrolysis, oligomerization and ClpP binding (Figure 6). More diverse regions appear to be the origin of species-specific activity. For example, both ECX and CCX contain the “IGF” motifs required for ClpP binding, but the area surrounding this region varies (Supplemental Figures 1, 2). This difference may explain the inability of CCX to interact with ECP in an *in vitro* setting. By contrast, the *Caulobacter* ClpX N-domain appears to support essential contacts required for *Caulobacter* viability that the *E. coli* N-domain does not provide. These contacts may include stringent recognition of substrates or interactions with critical adaptors needed for viability. We speculate that the differences in sequences between these species of N-domains (Supplemental Figures 1, 2) may underlie these different binding profiles.

The N-domain alters substrate targeting to ClpX by directly recognizing substrates or cooperating with a diverse set of adaptors for target degradation. In our study, we find fusing the *Caulobacter's* ClpX N-domain onto the AAA+ domain of *E. coli* ClpX restores the essential nature of ClpX in *Caulobacter*. We interpret this as evidence for the N-domain of the *Caulobacter* ClpX playing a unique role, such as facilitating degradation of the SocB toxin. However, differences between these N-domains do not result in purely exclusive behavior as the *E. coli* ClpX can support adaptor-dependent CtrA degradation and is able to restore growth defects in cells lacking SocB. In addition, an altered ability to process DnaX among the ClpX constructs suggest inherent differences in direct substrate recognition and may also reflect altered cooperation between the ClpX N-domain and AAA+ domain.

In conclusion, although the ClpX sequence is highly conserved between *E. coli* and *C. crescentus*, there are species-specific differences in activity that restrict the complementation between orthologs. These differences seem principally reflected by N-domain interactions, which account for both direct recognition and coordinated adaptor activity. However, it also seems that differences in substrate recognition by the ClpX AAA+ domain may affect how different ClpX orthologs support normal growth in *Caulobacter*. The work presented here argues that many aspects of ClpX function are conserved throughout bacterial evolution, but small differences may result in an altered ClpX specificity that is only critical in a particular species.

## Materials and methods

All *Caulobacter* strains, liquid or plated, were grown in PYE at 30°C, in the presence of the appropriate antibiotics or sugars.

### *In vitro* ClpX analysis

ClpX and ClpP from *C. crescentus* and *E. coli* were purified as before (Chien et al., 2007). Degradation of GFP-ssrA was performed as before (Rood et al., 2012).

### Caulobacter strains

Expression of ClpX variants driven by the *Caulobacter* ClpX promoter were generated by cloning 500 bp upstream of the Caulobacter clpX gene and fusing this to ClpX alleles using a pMR10-based vector. Plasmids were electroporated into Δ*socB, clpX::*Ω cells or parental strain UJ220 (Osteras et al., 1999). The following ClpX constructs were used: *Caulobacter* ClpX (CCX), *E. coli* ClpX (ECX), *Caulobacter* ClpX AAA+ domain (CCX minus the N-domain residues 2-53, ΔN-CCX), and the chimeric fusion of the *Caulobacter* N-domain substituted for the N-domain on the *E. coli* ClpX body, a direct N-terminal 2-53 aa substitution (CC-ECX).

### Caulobacter length analysis

Phase contrast images of *Caulobacter* cells (Zeiss AXIO ScopeA1) were subject to axial length analysis measuring pole-to-pole distance using the MicrobeJ software suite (ImageJ). Length is reported in microns.

### ClpX depletion

ClpX depletion was done in a similar fashion to (Bhat et al., 2013), except cells were back diluted twice during the ~20 h ClpX depletion. Samples for ClpX replete conditions were taken prior to depletion. Samples for both ClpX replete and depletion conditions were pelleted and snap frozen then re-suspended in an SDS loading buffer to a normalized OD600 = 0.1. Sample volumes were then heated at 95°C for 5 min. Equal volumes of sample were subjected to SDS-PAGE followed by Western transfer. Resulting blots were probed with anti-ClpX or anti-DnaX antibodies and visualized with appropriate secondary antibodies conjugated to HRP and chemifluorescent substrate.

### CtrA degradation

Δ*socAB* and Δ*socB, clpX::*Ω cells were diluted from overnight culture and allowed to reach mid-log phase, until the cells reached 0.3–0.5 OD600. Translational inhibitor chloramphenicol was added to a final concentration of 30 μg/ml. Following the addition of chloramphenicol, aliquots were removed every 30 min for 2 h. Cells were pelleted and snap frozen then re-suspended, normalized to an OD600 of 0.3. Sample volumes were heated at 95°C for 5 min. Equal volumes of sample were subjected to SDS-PAGE followed by Western transfer. Resulting blots were probed using an anti-CtrA antibody and visualized as above.

### Liquid growth assay

Δ*socAB* and Δ*socB, clpX::*Ω with the corresponding plasmids were grown from single colonies. For the time courses, samples were back diluted to a starting density of OD600 = ~0.1, and changes in optical density were measured over time. Resulting growth curves are the average of biological replicates, *n* = 3. Error bars represent standard deviation for the set of *n* = 3 (Figure 3D).

### Plated growth assays

Δ*socAB* and Δ*socB, clpX::*Ω with appropriate plasmids were grown from single colony into log growth. All plating samples started with a density of ~0.1 OD600 then followed a ten-fold dilution for each subsequent spot. Four microliters of resulting cultures was used to spot onto solid media and grown for ~3 days.

## Author contributions

RV and JN performed experiments. RV and PC designed experiments and wrote the manuscript.

### Conflict of interest statement

The authors declare that the research was conducted in the absence of any commercial or financial relationships that could be construed as a potential conflict of interest.
