# Supplementary material for: The Essential Role of ClpXP in Caulobacter crescentus Requires Species Constrained Substrate Specificity
Source: Front Mol Biosci. 2017 May 9;4:28. doi: 10.3389/fmolb.2017.00028 (PMC5422525; doi:10.3389/fmolb.2017.00028)

**Supplemental Information**

Supplemental Table 1. Strains used in this study.

| **strain** | **description** | **reference** |
| --- | --- | --- |
| UJ200 | CB15N *clpX*::spec,xylX:ClpX(tet) | Jenal et al., EMBO 1998 |
| CPC565 | UJ200 p*kanR (*pMR10) | this study |
| CPC566 | UJ200, pCCX | this study |
| CPC567 | UJ200, pECX | this study |
| CPC568 | UJ200, pΔN-CCX | this study |
| CPC569 | UJ200, pCC-ECX | this study |
| CAC219 | NA1000 *ΔsocAB* | C. Aakre/M. Laub (MIT) |
| CPC575 | ΔsocB, clpX::spec | gift from P. Viollier |
| CPC570 | *ΔsocB*, *clpX*::spec, pkanR (pMR10) | this study |
| CPC571 | *ΔsocB*, *clpX*::spec, pCCX | this study |
| CPC572 | *ΔsocB*, *clpX*::spec, pECX | this study |
| CPC573 | *ΔsocB*, *clpX*::spec, pΔN-CCX | this study |
| CPC574 | *ΔsocB*, *clpX*::spec, pCC-ECX | this study |

Supplemental Figure 1*.* The *E. coli* ClpX and *Caulobacter crescentus* ClpX share high homology (identical residues marked by asterisks, similar residues with colons). The N-domain is shown in a dashed box.


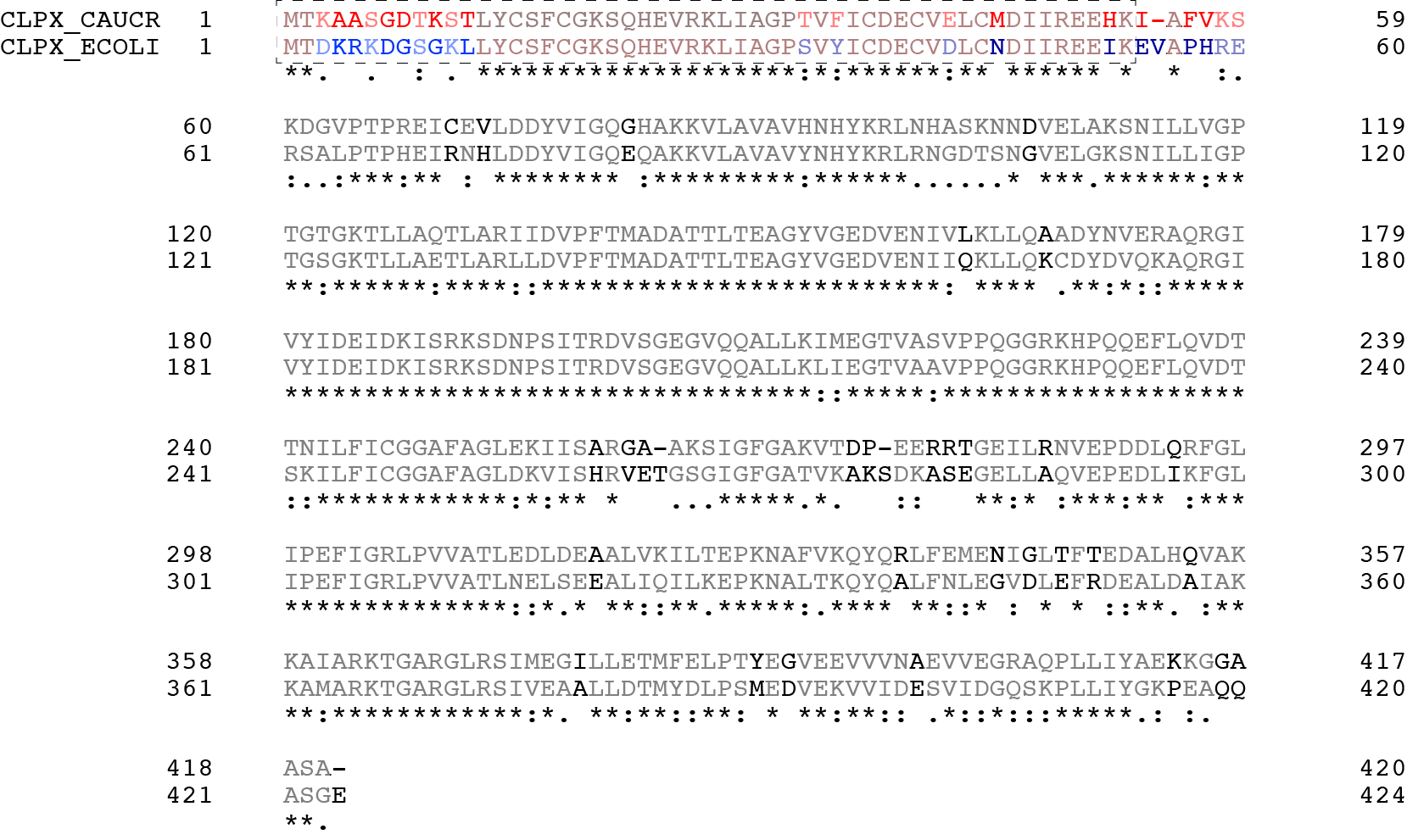


Supplemental Figure 2. An alignment of ClpX proteins (*C. crescentus,* *E. coli, M. tuberculosis, B subtilis, S. aureus* and *S. pneumoniae*) shows high conservation for amino acid residues constituting the N-domain. Blue shading highlights identical residues. The AAA+ domain also bears high sequence conservation, while the region surrounding the conserved IGF motif required for ClpP interaction is less similar. Yellow columns (labeled consensus) in inset denote conservation with larger numbers denoting higher conservation. Black columns in the full sequence (labeled consensus) denotes residue similarity across the protein.


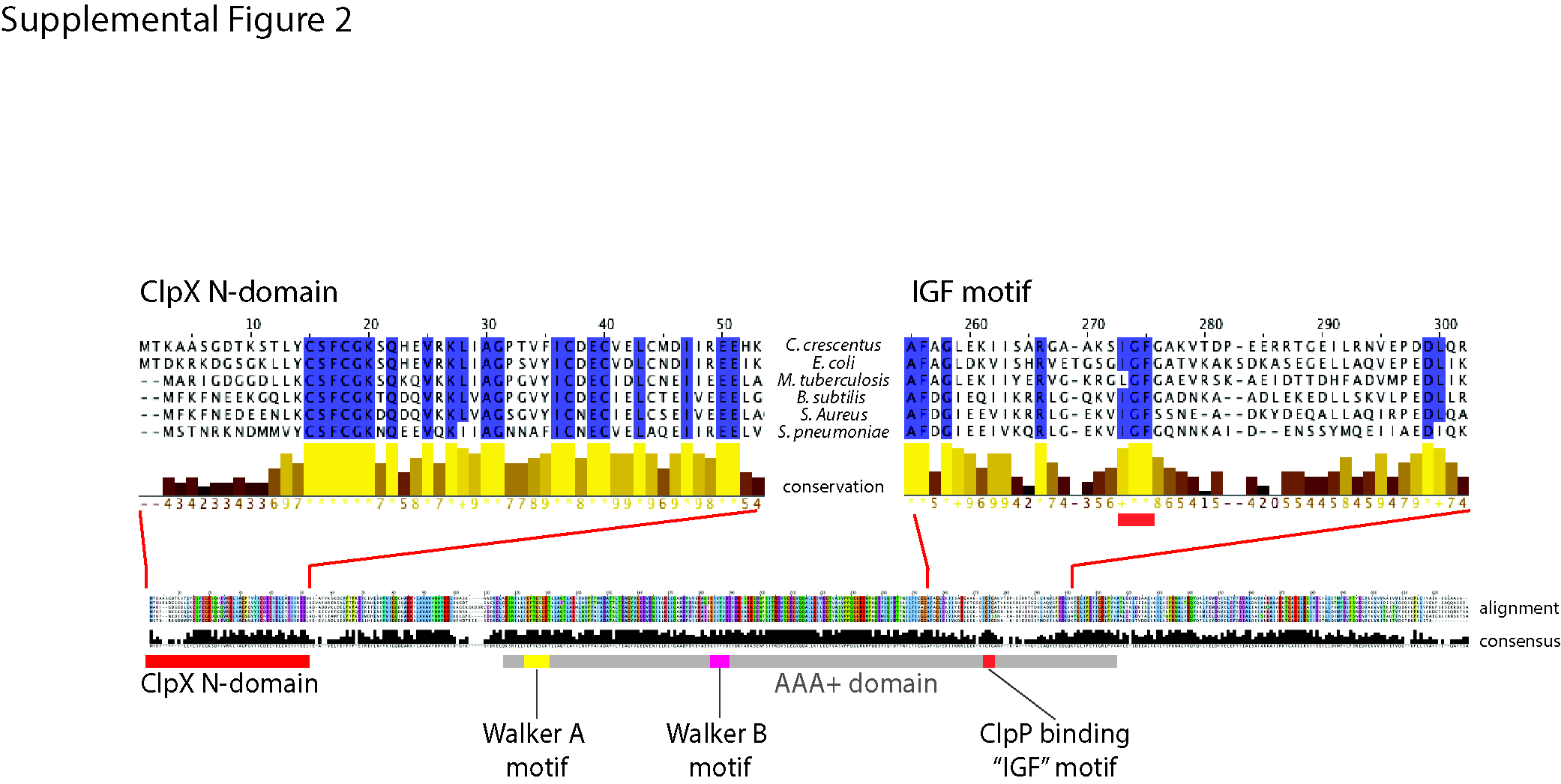


Supplemental Figure 3. (A) In support of Figure 2. Replicates show that expression of ECX reduces *Caulobacter* growth even in the presence (+xyl) of the CCX protein. (B) In support of Figure 3. Replicate dilution plating of Δ*socB* *clpX*::Ω *Caulobacter* strains expressing ClpX variants as labeled.


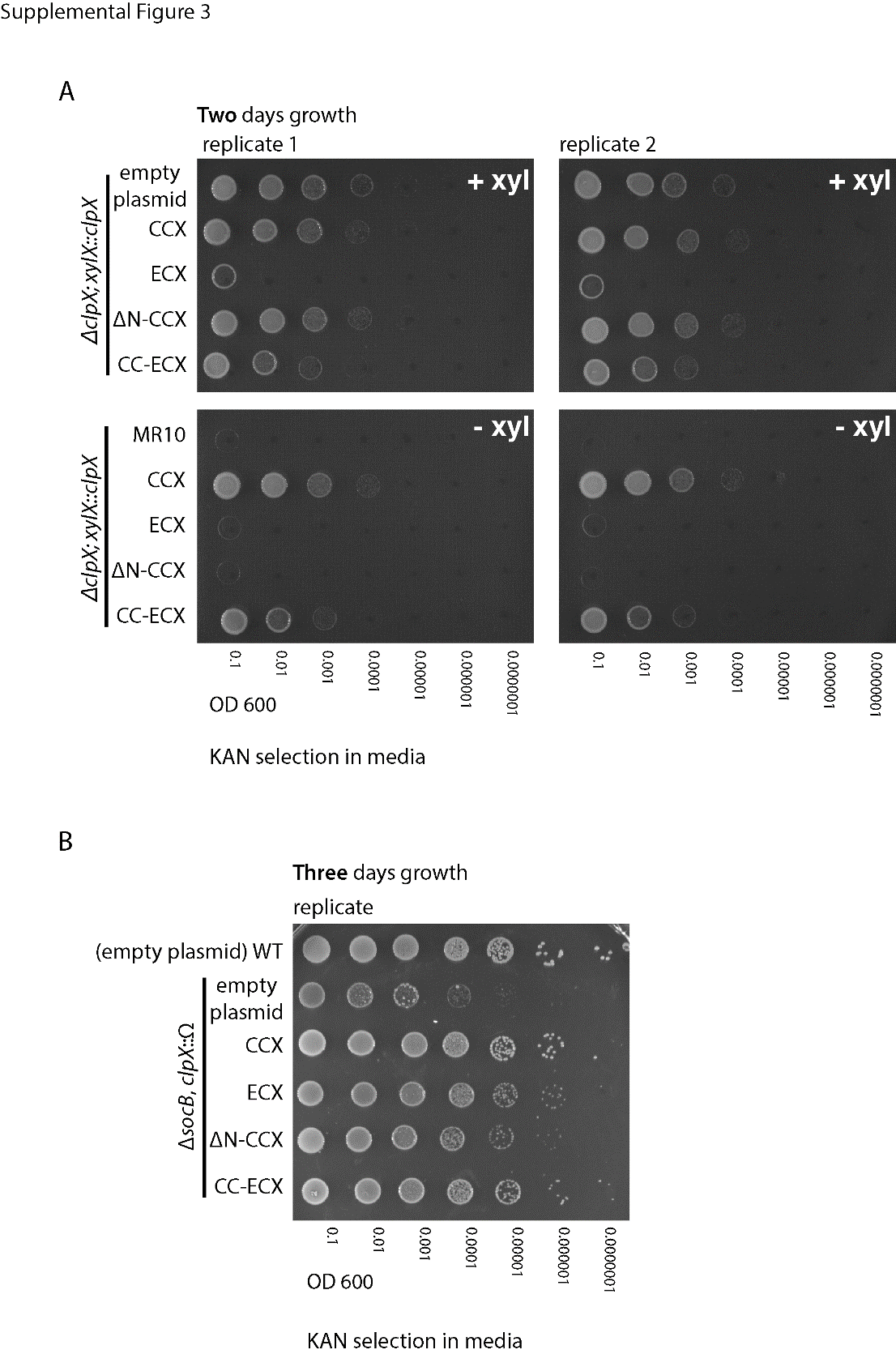


Supplemental Figure 4. In support of Figure 4. Replicate of anti-DnaX westerns of *ΔsocB, ΔclpX* cells with ClpX variants expressed as labeled. Similar to the blot shown in Figure 4, the steady state levels of full-length DnaX during logarithmic growth is higher in cells that lack the CCX N-domain compared to wildtype and cells expressing ECX alone fail to generate the γ2 form of DnaX.

**
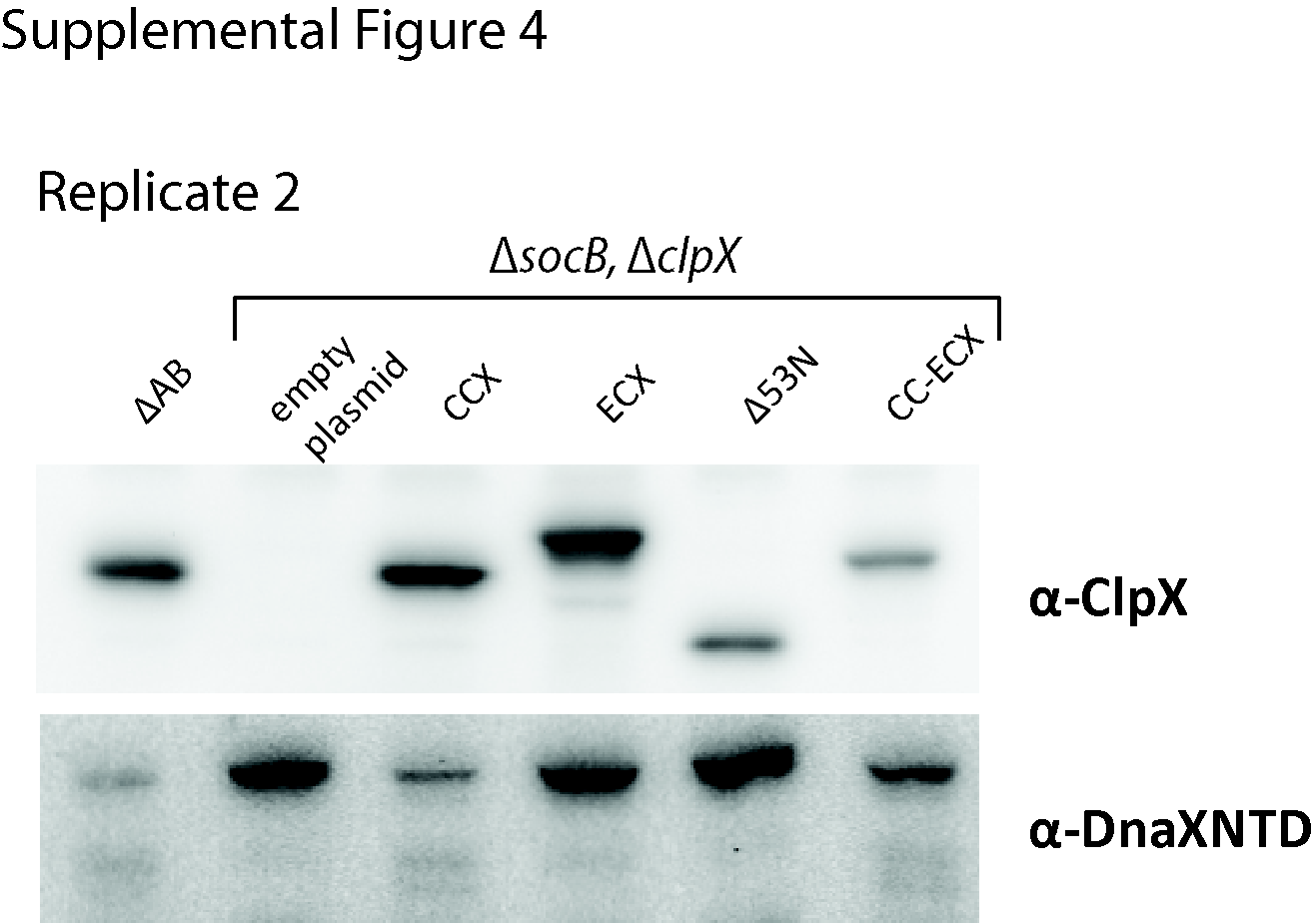
**

In support of Figure 5. (A) Replicates of the translational shutoff experiments. (B). CtrA levels were quantified relative to ClpP levels and plotted as log2 levels normalized to time zero. X-axis is time after antibiotic addition. The three independent slopes from the plots of each time course were averaged to determine the half-life and standard deviation for the table reported in Figure 5A.


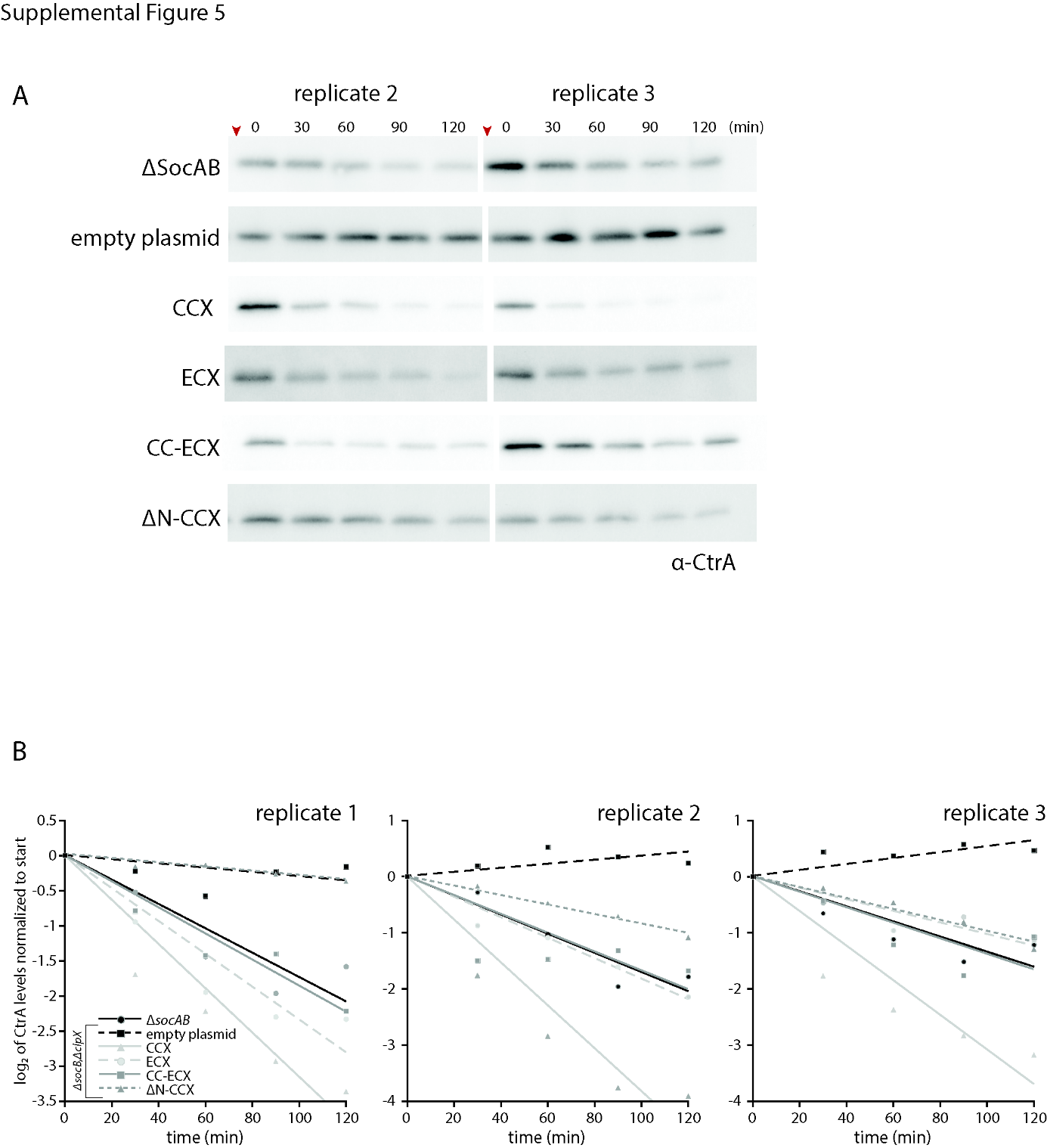

Supplement: Supplementary file 1 [file DataSheet1.docx]
